# The bidirectional relationship between depression and perceived social support among college freshmen: evidence from longitudinal and daily diary methods

**DOI:** 10.3389/fpsyg.2025.1676105

**Published:** 2025-12-17

**Authors:** Han Han, Xinyu Yu, Huxi Li, Nana Sun

**Affiliations:** 1 School of Psychology, Qufu Normal University, Qufu, China; 2 Department of Applied Psychology, Lyuliang University, Lyuliang, China

**Keywords:** depression, perceived social support, longitudinal design, daily diary method, freshmen

## Abstract

**Background:**

Depression significantly impairs adolescent mental health and adaptation, a critical issue for freshmen. Although depression and perceived social support (PSS) are known to be associated, their bidirectional relationship remains poorly understood.

**Methods:**

This study employed a multi-method approach to clarify this relationship. Study 1 was a three-wave longitudinal survey (*N* = 761) conducted over 3 months to examine trait-level associations. Study 2 utilized a 7-day daily diary method (*N* = 1,016) to assess state-level dynamics and minimize recall bias.

**Results:**

Cross-lagged analyses from the longitudinal study (Study 1) showed a reciprocal relationship: baseline depression predicted lower subsequent PSS, and baseline PSS predicted lower subsequent depression. Converging evidence from the daily diary study (Study 2) confirmed this bidirectional pattern at the daily level: daily state depression predicted lower PSS the next day, and conversely, daily PSS predicted lower state depression the next day.

**Conclusion:**

Our findings consistently demonstrate the bidirectional relationship between PSS and depressive symptoms across short-term and long-term timeframes. This underscores the importance of addressing both factors simultaneously in interventions aimed at enhancing the psychological adjustment and mental well-being of freshmen.

## 1 Introduction

Mental health among university students has garnered increasing attention in recent years, with depression emerging as a major psychological challenge in this population (Luo et al., 2025). Recent surveys indicate that approximately 29% of Chinese college students exhibit varying degrees of depressive symptoms, a figure that has shown a persistent upward trend in recent years (Li G. et al., 2023; Li R. et al., 2023). Clinically, depression is characterized by symptoms such as low mood, anhedonia, and cognitive sluggishness (Franz et al., 2021). Depression is commonly classified into two distinct forms: trait depression, which refers to stable, long-term depressive symptoms, and state depression, a transient emotional disturbance triggered by situational stressors (Merkitch et al., 2017; Chiappelli et al., 2014; Zhang et al., 2024). Both forms of depression negatively impact individuals’ quality of life (Li P. et al., 2024; Li Y. et al., 2024), social functioning (Lam and Saunders, 2024), and overall mental health (Simmonds-Buckley et al., 2021). Therefore, understanding the underlying mechanisms of depression is crucial for improving the mental health of university students.

Depression is influenced by a variety of factors, with PSS being one of the key determinants (Li G. et al., 2023; Li R. et al., 2023). PSS refers to the assistance, resources, and help an individual or group receives from their surrounding environment (Hou and Ma, 2024). It is a critical resource for college students in coping with stress (Luo et al., 2024) and can be divided into two types: objective social support and PSS (Furmark et al., 2009). Objective social support refers to the tangible resources and material assistance available to an individual when facing challenges, which are real, measurable, and perceptible (Wen et al., 2020). In contrast, PSS refers to the subjective feelings and evaluations of the support received from others during social interactions (Xia et al., 2012). This type of support is focused on the perception of being cared for, supported, and loved (Barrera et al., 1993), reflecting the emotional value an individual perceives from others in social interactions (Song and Zhang, 2016). PSS emerges in response to external events (Zhou et al., 2024). It plays a significant predictive role in mental health and also has positive effects on social relationships (Ye, 2005). PSS effectively alleviates psychological stress, maintains and improves mental health, and enhances quality of life (Xiao and Yang, 1987). Moreover, it serves as an important resource for improving life satisfaction (Yang and Ye, 2014). Conversely, a lack of PSS, combined with prolonged negative emotional states, can challenge individuals’ mental health (Seehuus and Peisch, 2021). Therefore, PSS is considered to have more psychological significance than objective social support (Xia et al., 2012).

Some researchers suggest that PSS may have a long-term predictive effect on depression. A longitudinal study by Birg et al. (2025) investigated the relationship between mental health symptoms and social functioning among university students and found that as PSS increased, the number of depressive symptoms decreased. Several longitudinal studies have also shown that higher levels of PSS significantly predict a reduction in future depression levels. Lakey and Cronin (2008) emphasized that PSS is consistently negatively correlated with depression, with this negative relationship remaining stable across time and contexts. In a three-wave longitudinal study with university students, Kleiman and Liu (2013) found that individuals with higher levels of PSS had significantly lower depressive symptoms 6 months later, and depression levels also slightly predicted subsequent PSS. According to the main-effect model, PSS itself directly enhances an individual’s emotional experience, well-being, and self-worth, thereby improving mental health and reducing depressive symptoms (House et al., 1988). PSS reduces depression through three core mechanisms: First, the Affective Pathway, where PSS makes individuals feel cared for, understood, and accepted, thereby enhancing their sense of belonging and security. This emotional support directly reduces feelings of loneliness and helplessness, countering negative emotions. Second, the Cognitive Pathway, where higher PSS helps individuals form a more positive worldview, thus reducing negative attribution and self-blame tendencies. Finally, the Behavioral Pathway, where individuals with PSS networks are more likely to adopt healthier lifestyles, which in the long term reduces depression risk (Lakey and Orehek, 2011). Therefore, this study hypothesizes that PSS has a long-term predictive effect on depression.

Additionally, a diary study by Wang et al. (2023) found that on days with more parental and peer support, adolescents experienced lower daily stress and negative emotions, and higher positive emotions. This confirms that adolescents with greater PSS from parents and peers exhibit less stress and more positive outcomes. Sliwinski et al. (2009) found that daily fluctuations in PSS are closely linked to changes in stress responses, with an increase in PSS leading to a reduction in psychological distress in the short term. Further, Yang et al. (2025) demonstrated through a diary method that daily fluctuations in PSS can predict changes in an individual’s well-being on the same or the following day. According to the social support buffering model (Cobb, 1976; Cohen and Wills, 1985), the buffering effect of PSS operates through multiple interconnected pathways, including psychological, behavioral, and physiological mechanisms, mitigating the adverse effects of stress and depression. In the psychological and cognitive mechanisms, PSS can change an individual’s cognitive appraisal of stress events, reduce threat perceptions, and enhance the sense of control over the situation (Lazarus and Folkman, 1984). On the behavioral level, PSS promotes adaptive coping behaviors and health-related behaviors, while reducing maladaptive coping behaviors such as avoidance or rumination (Uchino, 2004). In physiological and neurobiological mechanisms, PSS alleviates physiological stress responses by modulating the hypothalamic–pituitary–adrenal (HPA) axis and the autonomic nervous system (Mueller et al., 2022). Schiano Lomoriello et al. (2023) explored the impact of personal proxemics and empathy on depression from a neuroscience perspective, suggesting that individuals’ physical and social “closeness” may influence their experience of PSS, thereby impacting depressive symptoms. PSS plays a key moderating role in an individual’s ability to cope with stress. Specifically, as an external resource, PSS can weaken the negative impact of stress events on an individual’s emotional and psychological health. When individuals perceive higher levels of PSS, their subjective assessment of stress decreases, thus reducing negative emotional reactions and preventing an increase in depression levels (Cohen and Wills, 1985). Therefore, this study proposes the hypothesis that PSS has a short-term predictive effect on depression.

In addition, depression has a long-term predictive effect on PSS. A study by Mohr et al. (2004) on the mental health of patients with multiple sclerosis showed that depression is related to PSS, available social support, satisfaction with support, and emotional support needs. Karr and White (2024) examined whether there were differences in subjective cognitive issues and the use of cognitive strategies between university students with depression and those without. Their findings indicated that students with depression reported greater subjective cognitive concerns and poorer cognitive strategies compared to those without depression (Karr and White, 2024). Individual cultural and cognitive levels influence how social support is provided and interpreted, thereby affecting the quality and effectiveness of PSS (Altuncu et al., 2023). Recent longitudinal empirical studies have provided robust evidence for the pathway of “depression → cognitive decline.” For instance, a cross-lagged panel study by Wu et al. (2021) on elderly individuals with depression showed that baseline depressive symptoms could predict a decrease in cognitive function during follow-up. Cognition is an important predictor of PSS (Howie et al., 2023), and for adolescents, maladaptive cognitive schemas can reduce their ability to cope with negative events, thereby exacerbating depressive symptoms (Wong et al., 2020). According to the cognitive theory of depression, individuals with depression may exhibit cognitive biases, often perceiving themselves as incompetent, worthless, or flawed. They may think, “I am not good enough,” “I am a failure,” or “No one likes me” (Beck, 1967; Gittins and Hunt, 2020; Yuan et al., 2024), and are characterized by negative thinking and negative self-evaluations (Xu and Zhang, 2016). As a result, when depressed individuals encounter social support, they may perceive the support provided by others as incidental and unsustainable, rather than something they believe they deserve. Even when support is available, they may find it difficult to perceive or correctly interpret it, which could have long-term negative effects (Xuan et al., 2023). Based on this, the present study hypothesizes that depression has a long-term predictive effect on PSS. Depression may also exert a short-term predictive effect on PSS. Tu et al. (2025) investigated the relationship between anxiety and social avoidance among college students, along with the mediating roles of depression and interpersonal trust. The findings revealed that depression was positively associated with social avoidance, whereas interpersonal trust was negatively correlated with it, suggesting that individuals experiencing depression tend to withdraw socially and exhibit lower levels of trust in others. Horwitz et al. (2023) proposed that social withdrawal is linked to maladaptive psychological adjustment and reflects behavioral manifestations of internalized social anxiety or depressive affect. Using a seven-day diary method, Kelly et al. (2012) examined the associations among social safety, PSS, and maladaptive adjustment, confirming that poor social relationships negatively predict PSS, which can be explained by maladaptive adjustment.

According to social withdrawal theory, individuals with depressive symptoms tend to reduce social interactions, actively avoiding social situations and minimizing communication with others. Such withdrawal prevents them from fully experiencing care and support from family, friends, and colleagues, thereby diminishing their PSS. Moreover, they may overlook or misinterpret others’ supportive intentions and struggle to accurately recognize available social resources, consequently experiencing lower utilization and satisfaction with PSS (Rubin et al., 2009). Adolescence is a critical developmental stage for the emergence of social withdrawal, during which such behavioral patterns are more likely to form, influencing both social adaptation and mental health (Wood et al., 2022). Adolescents experiencing depression may feel powerless or avoidant in interpersonal situations and exhibit poor psychological adjustment, consequently experiencing a short-term decline in PSS. Based on these considerations, the present study hypothesizes that depression has a short-term predictive effect on PSS.

In summary, while previous research has explored the relationship between depression and PSS, several limitations remain. First, most studies have employed cross-sectional designs, which fail to capture the dynamic nature of the relationship between depression and PSS. For example, during the COVID-19 pandemic, some studies found a negative correlation between PSS and depression, anxiety, and insomnia. However, these findings were based on measurements taken at a single time point (Smith and Beaton, 2008). Second, many longitudinal studies involve long follow-up periods (Hill et al., 2016), typically spanning 6 months, a year, or even longer. Such extended timeframes make it difficult to capture subtle and rapid changes in psychological states and PSS over shorter periods, such as weeks or months. Finally, accurately recalling psychological states and PSS from the past few weeks or months requires participants to possess strong memory and recall abilities. If a participant’s current psychological state is poor, their ability to accurately recall past states may be compromised (Horwitz et al., 2023). Therefore, frequent short-term measurements can reduce recall bias and enhance the authenticity and sensitivity of the data (Cormack et al., 2024).

To address these issues, this study builds upon existing research and theory to further explore the bidirectional predictive relationship between depression and PSS in college students. Study 1 adopts a longitudinal tracking method with a one-month interval over 3 months to examine the relationship between depression and PSS. To overcome the common issue in existing research, where PSS is measured through retrospective recall—a method prone to recall bias—Study 2 employs a diary method to explore the short-term causal predictive relationship between state depression and PSS. This approach aims to minimize recall bias and enhance the ecological validity of the study (Knapp et al., 2021).

In conclusion, this research combines longitudinal tracking (Study 1) and diary methods (Study 2) to investigate the relationship between depression and PSS in college freshmen. Based on social cognitive theory and social withdrawal theory, Hypotheses H1 and H2 are proposed. Additionally, drawing on the main-effect model and buffering theory, Hypotheses H3 and H4 are put forward. The specific hypotheses are as follows:

Hypothesis H1: Pre-test depression negatively predicts post-test PSS.

Hypothesis H2: Previous state depression negatively predicts the next measurement of PSS.

Hypothesis H3: Pre-test PSS negatively predicts post-test depression.

Hypothesis H4: Previous PSS negatively predicts the next measurement of state depression.

## 2 Study 1: the effect of depression on PSS—evidence from a longitudinal design

Study 1 adopts a three-wave longitudinal tracking design to examine the long-term relationship between repeated assessments of depressive symptoms and PSS across 3 months.

### 2.1 Participants

A total of 1,040 university freshmen were recruited from a university and participated in three waves of survey tracking at one-month intervals. Data collection spanned from September to December 2024 during participants’ first academic year. This study followed a cumulative screening approach, and 66 data points were excluded due to incomplete participation (Schlomer et al., 2010). In addition, 187 responses were excluded due to incorrect answers to lie-detection items (Meade and Craig, 2012), and 36 data points were removed due to extreme values (Osborne and Overbay, 2004). An independent samples t-test comparing baseline data between study dropouts (*n* = 279) and retained participants (*n* = 761) revealed no significant differences in depression and PSS scores (all *p* > 0.05). Furthermore, a chi-square test indicated no significant gender differences between completers and non-completers [*χ*^2^(1) = 0.11, *p* > 0.05], suggesting that attrition was not systematically related to gender. The final valid sample consisted of 761 participants, which met the minimum statistical requirements and data screening standards (Kline, 2023). The participants’mean age was 18.21 ± 0.53 years, including 291 males and 470 females. All participants were informed that they could withdraw from the study at any time. Ethical approval was obtained from the local ethics committee (No. 2024132).

### 2.2 Instruments

#### 2.2.1 Depression scale

The Patient Health Questionnaire-8 (PHQ-8) is a revised version of the PHQ-9 designed to assess depressive symptoms. Developed by Kroenke et al. (2009), it excludes the suicide-related item from the PHQ-9, retaining eight items that evaluate the frequency and severity of depressive symptoms over the past 2 weeks. Moehring et al. (2021) tested the longitudinal measurement invariance of the PHQ-8 across multiple time points (2, 4, 6, and 12 months) and across different populations, confirming its comparability over time and supporting its use in longitudinal follow-up studies to track symptom changes. Each item is rated on a 4-point scale, where 0 indicates “not at all” and 4 indicates “nearly every day.” Higher scores indicate greater levels of depression. In this study, the Cronbach’s alpha coefficients for the PHQ-8 at the three time points were 0.84, 0.87, and 0.88, respectively.

#### 2.2.2 Perceived social support scale

The Perceived Social Support Scale (PSSS) was developed by Dahlem et al. (1991) and later translated and revised by Jiang (2001). This study used the revised version. The scale employs a 7-point Likert response format, where 1 represents “strongly disagree” and 7 represents “strongly agree.” Higher scores indicate higher levels of PSS. The Cronbach’s alpha coefficients for the PSSS at the three time points were 0.93, 0.95, and 0.96, indicating excellent internal consistency.

### 2.3 Data analysis methods

**Figure 1:**
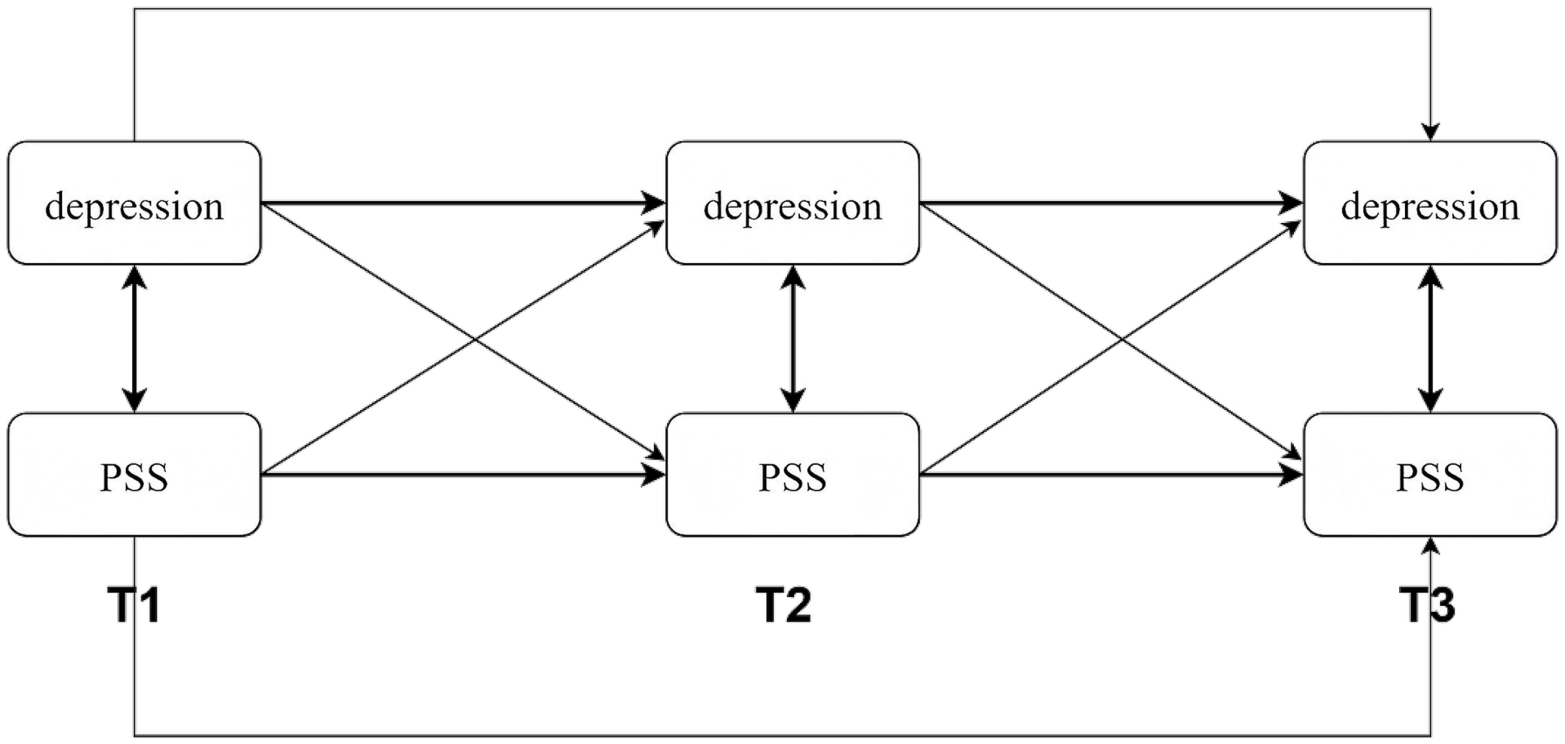
Cross-lagged model of depression and PSS.

Data analysis for Study 1 was conducted using SPSS 27.0 and Mplus 8.0. Descriptive statistics, correlation analyses, and common method bias tests were first performed using SPSS 27.0. Based on the results of the correlation analyses, a cross-lagged structural equation model was constructed in Mplus 8.0 to examine the bidirectional relationship between depression and PSS (see Figure 1).

Model 1 (M1) served as the baseline autoregressive model, establishing the temporal stability of both constructs. Model 2 (M2), the forward causation model, extended M1 by incorporating cross-lagged paths from prior depression to subsequent PSS. Model 3 (M3), the reverse causation model, extended M1 by adding paths from prior PSS to subsequent depression. Finally, Model 4 (M4), the reciprocal model, included all autoregressive and cross-lagged paths to simultaneously test for bidirectional predictive relationships between depression and PSS.

### 2.4 Common method bias test

Given the reliance on self-report measures, we assessed the potential for common method bias using Harman’s single-factor test following established procedures (Zhou and Long, 2004). Exploratory factor analysis yielded six factors with eigenvalues exceeding 1. The first unrotated factor explained 30.11% of the total variance, falling below the recommended threshold of 40%. These results suggest that common method bias does not substantially threaten the validity of our findings.

### 2.5 Preliminary results

Table 1 displays the means, standard deviations, and correlation coefficients for all study variables across the three assessment waves. As hypothesized, depression showed significant negative correlations with PSS at each time point. These consistent cross-sectional associations provide initial support for our central hypothesis of an inverse relationship between depression and PSS.

**Table 1:** Means, standard deviations, and correlation matrix of depression and PSS (*N* = 761).

Variable	M	SD	1	2	3	4	5	6
1. T1 Depression	1.78	0.50	1					
2. T1 PSS	5.27	1.04	−0.43^**^	1				
3. T2 Depression	1.81	0.54	0.71^**^	−0.38^**^	1			
4. T2 PSS	5.21	1.05	−0.38^**^	0.77^**^	−0.36^**^	1		
5. T3 Depression	1.81	0.54	0.65^**^	−0.36^**^	0.70^**^	−0.36^**^	1	
6. T3 PSS	5.19	1.03	−0.41^**^	0.70^**^	−0.38^**^	0.77^**^	−0.43^**^	1

T1, T2, and T3 represent the three measurement time points. *p* < 0.01.

We also evaluated the autoregressive and cross-lagged models of depression and PSS. According to the fit criteria outlined by Hu and Bentler (1998), a model is considered a good fit when TLI > 0.90, CFI > 0.90, SRMR < 0.10, and RMSEA < 0.08. The fit statistics for all models are presented in Table 2.

**Table 2:** Model fit indices.

Model	*χ* ^2^	*df*	*p*	RMSEA	SRMR	CFi	TLi	Model comparison	Δχ^2^	Δ*df*
M1	33.56	6	0.00	0.08	0.06	0.98	0.96			
M2	19.82	4	0.00	0.07	0.04	0.99	0.97	M1-M2	13.74	1
M3	17.29	4	0.00	0.07	0.03	0.99	0.97	M1-M3	16.27	1
M4	5.76	2	0.06	0.05	0.01	0.99	0.98	M1-M4	27.80	2

RMSEA, Root Mean Square Error of Approximation; SRMR, Standardized Root Mean Square Residual; CFI, Comparative Fit Index; TLI, Tucker–Lewis Index.

**Figure 2:**
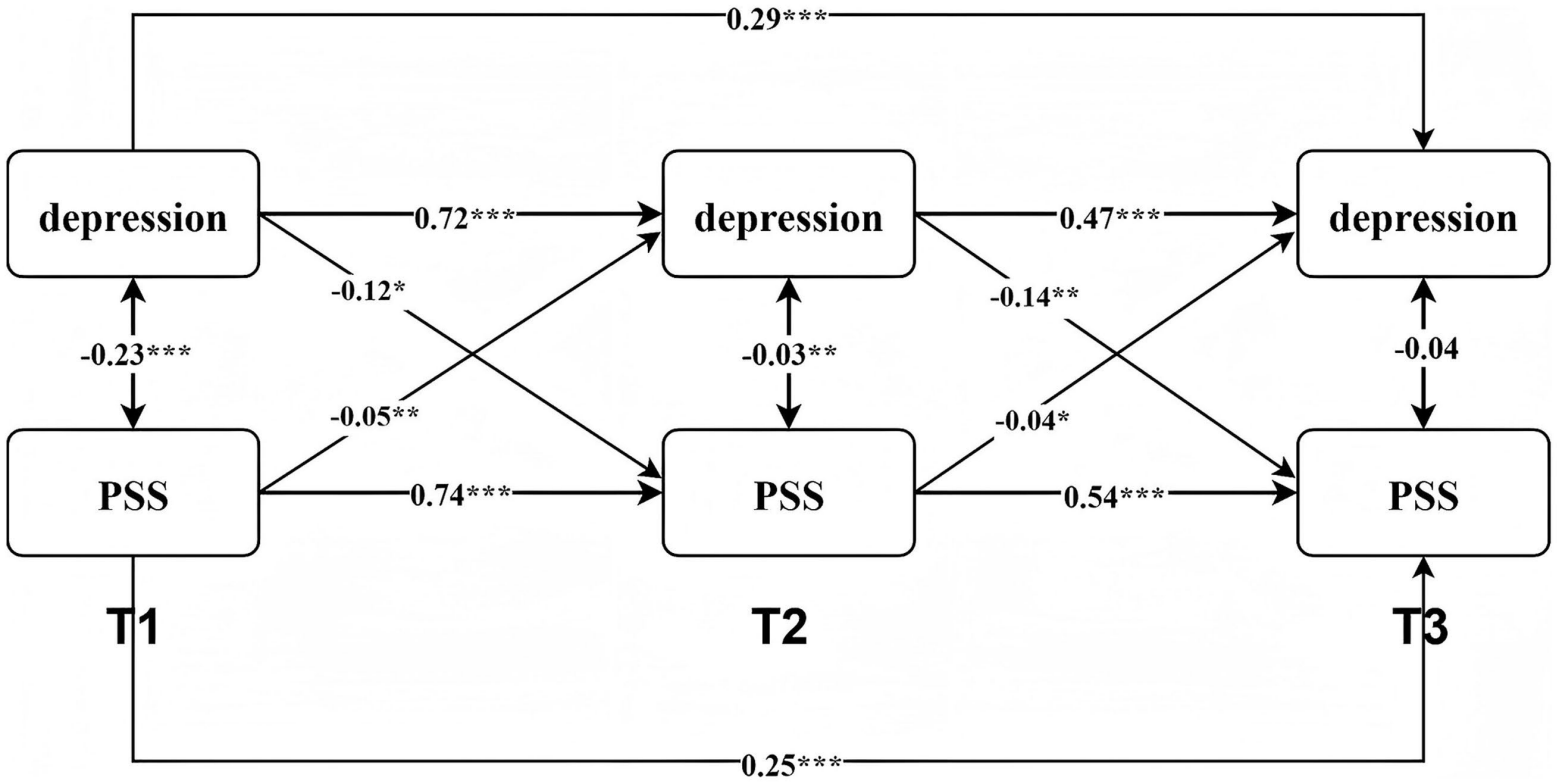
Cross-lagged model diagram of depression and PSS.

The cross-lagged relationships between depression and PSS are presented in Figure 2. The autoregressive model (M1) demonstrated good model fit. In this model, the stability coefficients for both depression and PSS were statistically significant, reflecting substantial temporal stability in both constructs across measurement waves (see Table 3).

**Table 3:** Standardized autoregressive and cross-lagged estimates.

Model	Autoregressive path	β	95% CI	Cross-Lagged Path	β	95% CI
M4	PHQ _T1_ → PHQ _T2_	0.668***	[0.614, 0.723]	PHQ _T1_ → PSS _T2_	−0.096***	[−0.155, −0.037]
	PHQ _T2_ → PHQ _T3_	0.475***	[0.385, 0.593]	PHQ _T2_ → PSS _T3_	−0.071*	[−0.129, −0.013]
	PHQ _T1_ → PHQ _T3_	0.277***	[0.183, 0.371]			
	PSS _T1_ → PSS _T2_	0.740***	[0.697, 0.783]	PSS _T1_ → PHQ _T2_	−0.060*	[−0.115, −0.004]
	PSS _T2_ → PSS _T3_	0.544***	[0.512, 0.650]	PSS _T2_ → PHQ _T3_	−0.074**	[−0.142, −0.006]
	PSS _T1_ → PSS _T3_	0.256***	[0.191, 0.321]			

T1 = T1, T2, and T3 represent the three measurement time points; β = standardized coefficient. *p < 0 0.05, **p < 0 0.01, ***p < 0 0.001.

The forward causation model (M2) also exhibited good fit to the data. Model comparisons indicated that M2 provided significantly better fit than M1: Δ*χ^2^*(1, 761) = 13.74, *p* < 0.001. The cross-lagged path from prior depression to subsequent PSS was significant, indicating that depression negatively predicts later PSS.

The reverse causation model (M3) showed further improvement in model fit. Compared to M2, M3 demonstrated significantly better fit: Δ*χ^2^*(1, 761) = 16.27, *p* < 0.001. The path from prior PSS to subsequent depression was significant, demonstrating that PSS negatively predicts later depression.

Finally, the reciprocal model (M4) showed the best overall fit among the competing models: Δ*χ^2^*(1, 761) = 27.80, *p* = 0.06. In this final model, depression at earlier time points negatively predicted subsequent PSS, while prior PSS also negatively predicted later depression. These results collectively support a bidirectional predictive relationship between depression and PSS.

In the three-wave longitudinal data of this study, both the autoregressive and cross-lagged paths between depression and PSS demonstrated high stability. The autoregressive coefficients showed minimal variation across time points, indicating consistent temporal continuity in both depression and PSS levels. To formally test the stability of cross-lagged effects, we constrained the core path coefficients (autoregressive and cross-lagged paths) to be equal across time intervals. The constrained model demonstrated good fit to the data (CFI = 0.948, TLI = 0.909, SRMR = 0.043). Parameter estimates further confirmed high stability across measurements, with all cross-time path coefficients showing strong consistency in both magnitude and direction. Specifically, the paths from depression to subsequent PSS (*β* = −0.079 vs. -0.084) and from PSS to subsequent depression (*β* = −0.104 vs. -0.102) were nearly identical. Similarly, autoregressive paths remained stable across time (PHQ: *β* = 0.647 vs. 0.665; PSS: *β* = 0.730 vs. 0.737). These results indicate that the relationships between variables remained constant throughout the study period, satisfying the assumption of longitudinal invariance and ensuring the validity of cross-temporal comparisons.

The findings overall support our hypothesis of model stability, demonstrating that the dynamic associations between depression and PSS remained relatively consistent across the study timeframe. Furthermore, we observed some asymmetry in the cross-lagged effects between depression and PSS. Such asymmetry is consistent with previous longitudinal and intensive longitudinal research, which has shown that temporal predictive relationships between psychological constructs are often influenced by their stability, baseline levels, and context sensitivity (Hamaker et al., 2015). Specifically, depression as a relatively stable emotional tendency demonstrates strong temporal autocorrelation, while PSS is more context-dependent and susceptible to short-term fluctuations in daily experiences. Consequently, the predictive path from depression to PSS may demonstrate greater stability or significance compared to the reverse pathway. This observed asymmetry does not reflect a model limitation but rather captures the inherent dynamic characteristics of psychological constructs across time.

In summary, Study 1 results demonstrate that prior depression negatively predicts subsequent PSS, and prior PSS negatively predicts subsequent depression. These findings support a long-term bidirectional predictive relationship between depression and PSS.

## 3 Study 2: the impact of state depression on PSS—evidence from a daily diary study

With the aim of reducing retrospective bias in assessing the relationship between depression and PSS, Study 2 adopted a daily diary method to explore their predictive relationship.

### 3.1 Participants

In Study 2, we recruited 1,032 university freshmen in 2024 for a 7-day daily diary study, following established research protocols (Li Y. et al., 2024). To account for time effects, participants were asked to complete the questionnaire at the same time each day, between 7:00 p.m. and 12:00 a.m. Participant attrition was primarily due to timing issues and the psychological burden associated with daily completion, as well as conflicts with academic or personal schedules. The attrition rate in this study was consistent with those observed in previous diary studies (Bolger et al., 2003; Conner and Lehman, 2012).

Ultimately, valid data from 1,016 participants were obtained, including 396 males and 620 females, with an average age of 18.21 ± 0.58 years. After replacing missing values with 999, a total of 7,112 data points were collected. This study was approved by the local ethics committee (No. 2024132).

### 3.2 Measures

#### 3.2.1 Daily depression

To reduce participant burden in the daily diary study, two items with high factor loadings from the depression scale used in Study 1 were adapted to measure state depression (Ye et al., 2023). The original items, “I have little interest or enjoyment in doing things” and “I feel down, depressed, or hopeless,” were modified to “I had little energy or interest in doing things today” and “I felt down, depressed, or hopeless today.” In this study, the Cronbach’s *α* coefficients for within-subject and between-subject reliability were 0.58 and 0.95, respectively.

#### 3.2.2 Daily perceived social support

The version of the Perceived Social Support Scale used in Study 2 was adapted for the diary method. The item “I felt understood and cared for by others today” was selected to measure participants’ daily PSS. The Cronbach’s α coefficients for within-subject and between-subject reliability were 0.52 and 0.92, respectively (Knapp et al., 2021). Many daily diary studies use simplified items to measure depression and PSS (Fisher et al., 2018).

As diary-based measurements often involve fewer items and exhibit greater within-day variability, within-subject Cronbach’s α is typically lower than between-subject reliability (Cranford et al., 2006). In this study, the within-day α for state depression and daily PSS were 0.58 and 0.52, respectively. While these values are relatively low, they remain within the acceptable range for diary studies.

### 3.3 Procedure

Questionnaires were distributed and collected via an online platform. Before the formal data collection, participants provided demographic information. For seven consecutive days, links to the daily questionnaire were sent out at 5:00 p.m., and responses were accepted until midnight (12:00 a.m.). No delayed or make-up submissions were allowed.

### 3.4 Data analysis

Data analysis was conducted with SPSS 27.0 and Mplus 8.0. Missing values, represented by 999, were managed through full information maximum likelihood estimation (FIML). Because the daily observations (*N* = 7,112) were nested within individuals (*N* = 1,016), the data comprised two levels: within-person (Level 1) and between-person (Level 2).

Initially, a null model (lacking predictors) was formulated to assess mean levels, within-person and between-person variances, correlations, and the intraclass correlation coefficient (ICC).

To further investigate the dynamic interaction between depression and PSS, this study adopted the framework proposed by Ye et al. (2023) and applied Dynamic Structural Equation Modeling (DSEM) to build a hierarchical regression model. The model included two levels: the within-person level (Level 1) and the between-person level (Level 2), with depression as the independent variable and PSS as the dependent variable (At the same time, PSS was treated as the independent variable and depression as the dependent variable for validation, with the results presented later in the text). Random intercepts and random slopes were computed for depression, PSS, and the time variable. Adhering to the longitudinal research framework (Cole and Maxwell, 2003), time was treated as a covariate at Level 1 to control for linear temporal trends.” The time variable was coded in accordance with the sequential order of data collection (Day 1 = 1, Day 2 = 2, etc.). Prior to analysis, depression, PSS, along with time, were all centered around their respective group means.

The given model specification is as follows:

At Level 1 (within-person level), the PSS y_ij_ for individual i on day j modeled as.

Within-Person Level (Level 1):

$y_{\mathrm{ij}}=(\mathrm{PSS})=\beta_{0i}+\beta_{1i}(X_{\mathrm{ij}}-\bar{X})+\beta_{2i}(T_{\mathrm{ij}}-\bar{T})+r_{\mathrm{ij}}$

Where:

$\beta_{0i}$ : individual intercept (mean PSS).

$\beta_{1i}$ : effect of daily depression (person-mean centered).

$\beta_{2i}$ : effect of time (person-mean centered).

$X_{\mathrm{ij}}$ : observed daily depression.

$T_{\mathrm{ij}}$ : measurement day (assigned as Day 1 = 1, Day 2 = 2, etc.)

$r_{\mathrm{ij}}$ : residual variance at the observation level.

This centering approach distinguishes trait-level (between-person) from state-level (within-person) effects.

Between-person Level (Level 2):

At the between-person level, the model included random intercepts and slopes:


$$\beta_{0i}=\gamma_{00}+\mu_{0i}$$

$$\beta_{1i}=\gamma_{01}+\mu_{1i}$$

$$\beta_{2i}=\gamma_{02}+\mu_{2i}$$

Where $\gamma_{00}$ , $\gamma_{01}$ , and $\gamma_{02}$ are fixed effects, and $\mu_{0i}$ , $\mu_{1i}$ and $\mu_{2i}$ are person-level residuals (random effects).

In this model, $\beta_{0i}$ describes the random reference point at Level 1, while $\beta_{1i}$ and $\beta_{2i}$ denote the random coefficients at Level 1. The parameters $\gamma_{00}$ , $\gamma_{01}$ , and $\gamma_{02}$ represent the corresponding fixed effects (intercepts), and $\mu_{0i}$ , $\mu_{1i}$ , and $\mu_{2i}$ are the residuals associated with each respective equation.

**Figure 3:**
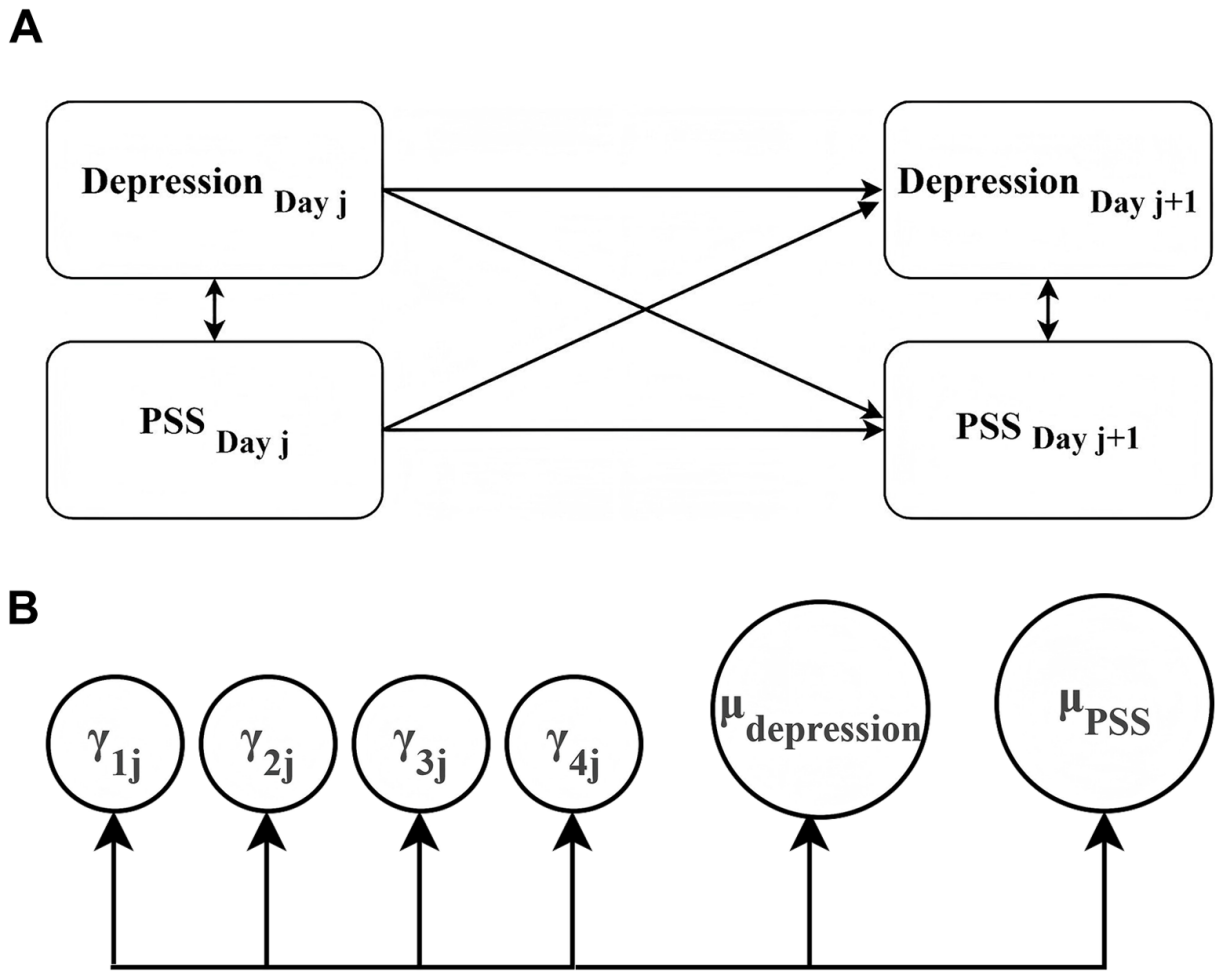
**(A)** Within-person effect. **(B)** Between-person effect.

To further explore relationships, a multilevel cross-lagged path model was constructed to examine day-to-day predictive associations between depression and PSS. As shown in Figure 3, *γ*_1j_and γ_4j_ represent autoregressive effects,γ_2j_ and γ_3j_ represent cross-lagged effects, μ_depression_ and μₚₛₛ reflect the intercepts for depression and PSS, respectively. In this model, both the intercept and slopes were specified as random. To improve clarity, the equations at the within-person level are omitted. The model specifications are provided below:


$$\begin{array}{l} \gamma_{\mathrm{ij}}({\text{Depression}}_{\text{Dayj}+1})=\gamma_{0j}+\gamma_{1j}({\text{Depression}}_{\text{Dayj}})\\ +\gamma_{2j}({\mathrm{PSS}}_{\text{Dayj}})+r_{\mathrm{ij}} \end{array}$$

$$\gamma_{\mathrm{ij}}({\mathrm{PSS}}_{\text{Dayj}+1})=\gamma_{0j}+\gamma_{3j}({\text{Depression}}_{\text{Dayj}})+\gamma_{4j}({\mathrm{PSS}}_{\text{Dayj}})+r_{\mathrm{ij}}$$

Among the parameters, *γ*_1j_ (previous-day depression) represents the slope indicating how participants’ depression on the previous day predicts depression on the following day.γ_2j_ (previous-day PSS) refers to the slope for how PSS on the previous day predicts depression on the next day. γ_3j_(previous-day depression) represents the slope for how depression on the previous day predicts PSS on the following day, and γ_4j_ (previous-day PSS) reflects the slope for how PSS on the previous day predicts PSS on the next day.

### 3.5 Results

#### 3.5.1 Descriptive statistics

Table 4 presents the means, within- and between-subject variances, ICC, and within- and between-subject correlations for depression and PSS. The results show ICC values of 0.511 for depression and 0.515 for PSS, indicating that 48.9% of the variance in depression and 48.5% of the variance in PSS were attributable to within-person fluctuations. The substantial between-person variance components confirm the stability of both constructs across individuals.

**Table 4:** Descriptive statistics and ICC.

Variable	M	Variance		ICC	Depression	PSS
		Within-person variance	Between-person variance			
Depression	1.738	0.212	0.222	0.511	1	−0.502^***^
PSS	7.363	2.155	2.291	0.515	−0.113^***^	1

The lower-left triangle presents within-person correlations, and the upper-right triangle presents between-person correlations. ***p < 0.001.

These findings demonstrate that daily fluctuations in depression and PSS occur at both within-person and between-person levels, with significant individual differences observed. The heterogeneity suggests our sample included individuals with relatively stable mental states alongside those experiencing more variable depressive states. This pattern of variability aligns with previous research documenting substantial within-person and between-person differences in daily emotional experiences and PSS (Nezlek, 2012; Zautra et al., 2005), thus supporting the validity of our measurement approach. The variance composition supports the use of multilevel modeling for subsequent analyses (Zhang et al., 2003). At the within-person level, correlation analysis revealed a significant negative relationship between depression and PSS (*r* = −0.113, *p* < 0.001), indicating that on days when individuals reported higher depression levels, they concurrently reported lower PSS.

#### 3.5.2 Multilevel regression analysis

To further examine the relationship between depression and PSS, a multilevel regression model was specified with depression as the independent variable and PSS as the dependent variable. Results showed that depression negatively predicted PSS (*γ* = −0.369, SE = 0.053, *t* = −6.991, *p* < 0.001). To ensure the accuracy of the results, this study further examined the relationship by treating PSS as the independent variable and depression as the dependent variable. A significant bidirectional predictive effect was found between PSS and depression. In the within-group analysis, depression showed a significant negative correlation with PSS (*γ* = −0.076, *p* < 0.001). In the between-group analysis, the negative correlation between PSS and depression remained significant (*γ* = −0.357, *p* < 0.001), indicating notable differences in PSS and depression across individuals. This further supports the bidirectional relationship between depression and PSS.

The individual mean centering method is used to handle time variables, primarily in the context of a multilevel model framework (where repeated observations are nested within individuals). This approach helps to more clearly distinguish between within-person effects and between-person effects.

This method allows for the separation of an individual’s time-varying fluctuations (e.g., emotional fluctuations due to daily experiences) from stable individual differences (e.g., a generally more pessimistic tendency). By centering individual values around their mean, it provides a clearer picture of how each individual’s temporal variations relate to their overall stable traits(Enders and Tofighi, 2007).

#### 3.5.3 Multilevel cross-lagged analysis

The results of the multilevel cross-lagged path analysis showed that depression from the previous day significantly positively predicted depression on the following day (*γ* = 0.198, *SD* = 0.023, *p* < 0.001, 95% *CI* = [0.153, 0.239]), and PSS from the previous day significantly positively predicted PSS on the following day (*γ* = 0.261, *SD* = 0.020, *p* < 0.001, 95% *CI* = [0.218, 0.299]). These findings suggest that both depression and PSS exhibit high within-person stability over a short 7-day period. Furthermore, depression from the previous day significantly negatively predicted PSS on the following day (*γ* = −0.305, *SD* = 0.071, *p* < 0.001, 95% *CI* = [−0.435, −0.168]), and PSS from the previous day significantly negatively predicted depression on the following day (*γ* = −0.021, *SD* = 0.006, *p* < 0.001, 95% *CI* = [−0.031, −0.010]). These results indicate a significant bidirectional predictive relationship between depression and PSS. Detailed results are shown in Table 5 below.

**Table 5:** The relationship between depression and PSS.

Effect	Variable	Fixed effect		Random effect	
		γ (SD)	95% CI	τ (SD)	95% CI
Intercept	PHQ	1.584^***^ (0.024)	[1.536, 1.629]	0.221^***^ (0.016)	[0.193, 0.252]
	PSS	7.512^***^ (0.076)	[7.367, 7.665]	1.780^***^ (0.147)	[1.550, 2.101]
Autoregressive effect	PHQ _T1_ → PHQ _T2_	0.198^***^ (0.023)	[0.153, 0.239]	0.123^***^ (0.013)	[0.102, 0.149]
	PSS _T1_ → PSS _T2_	0.261^***^ (0.020)	[0.218, 0.299]	0.126^***^ (0.012)	[0.103, 0.151]
Cross-lagged effect	PHQ _T1_ → PSS _T2_	−0.305^***^ (0.071)	[−0.435, −0.168]	0.917^***^ (0.147)	[0.657, 1.230]
	PSS _T1_ → PHQ _T2_	−0.021^***^ (0.006)	[−0.031, −0.010]	0.003^***^ (0.001)	[0.002, 0.005]

T1 represents the previous day, T2 represents the following day, SD stands for standard deviation; 95% CI represents the 95% credible interval for Bayesian estimates, ***p < 0.001.

To ensure the accuracy of the results, this study further examined the relationship by treating PSS as the independent variable and depression as the dependent variable. A significant bidirectional predictive effect was found between PSS and depression. In the within-group analysis, depression showed a significant negative correlation with PSS (*γ* = −0.076, *p* < 0.001). In the between-group analysis, the negative correlation between PSS and depression remained significant (*γ* = −0.357, *p* < 0.001), indicating notable differences in PSS and depression across individuals. This further supports the bidirectional relationship between depression and PSS.

## 4 Discussion

This study combined the tracking method and the diary method to explore the bidirectional predictive relationship between depression and PSS using different approaches. Study 1 found that depression at baseline significantly negatively predicted PSS at follow-up, while PSS at baseline also significantly negatively predicted depression at follow-up. Study 2 further revealed that, over a 7-day period, state depression on one day negatively predicted PSS on the following day, and PSS on one day also negatively predicted state depression on the following day. These findings suggest a bidirectional negative influence between depression and PSS, creating a vicious cycle.

The conclusions of this study complement and expand existing theories on depression and PSS, providing effective evidence for the adaptation process of college freshmen entering a new environment. The following sections will discuss the results in detail.

Firstly, Study 1 examined the bidirectional predictive relationship between depression and PSS. The results of Study 1 demonstrated a bidirectional negative predictive relationship between depression and PSS over three months, validating hypotheses H1 and H3. Specifically, higher depression predicted lower PSS, and vice versa. Previous studies have also shown a strong correlation between depression and social support, which is consistent with the results of Study 1 (Wang et al., 2025). However, research specifically focusing on PSS is limited. This study extends the research on social support, further revealing the important role of cognitive schemas related to social support in the dynamic prediction of depression. By focusing on the cognitive level, this research lays the foundation for better integrating social psychology and cognitive fields in future studies.

Moreover, in the bidirectional negative prediction between depression and social support, maladaptive cognitive schemas during the transition to university play a crucial role. For college freshmen, entering the university means leaving their familiar environment and the care of their parents, facing new pressures and challenges (Arnstein, 1980). This stage represents a critical period of transition from adolescence to adulthood, during which emotional fluctuations are sensitive (March-Llanes et al., 2017), and depression is prevalent (World Health Organization, 2021; Yao et al., 2019). Freshmen with high depression face greater challenges in adapting to the new environment. Specifically, individuals with depression often exhibit negative cognitive patterns, which hinder their adaptation to the new social environment and make it difficult for them to accurately perceive the support provided by others (Beck, 1967). This is consistent with the Cognitive Resource Depletion Hypothesis, which suggests that the emotional regulation and rumination of depressed individuals consume limited cognitive resources, impairing their executive function in a new environment and making it difficult for them to perform adaptation tasks (Elhai et al., 2021). This cognitive bias leads them to perceive lower levels of social support over time.

Furthermore, Gritti et al. (2024), in their study of predictors of poor mental health among healthcare workers during the pandemic, revealed that PSS can protect the psychological health of individuals in high-stress occupations. The role of PSS in alleviating depression is not only applicable to college freshmen but can also extend to other populations, supporting a broader psychological mechanism.

Moreover, depressive moods may weaken an individual’s motivation and ability to engage in social interactions, leading to increased avoidance behaviors and fewer opportunities to receive support. This aligns with previous research (Cacioppo and Hawkley, 2009). On the other hand, individuals with high PSS are more likely to receive support and strength from interpersonal relationships, such as those with parents and friends. When facing challenges, they tend to display relatively stable positive tendencies. This result can be explained by the main effect model, where social support provides positive feedback, increases self-efficacy, and helps individuals gain confidence and energy, thereby reducing depressive symptoms (House et al., 1988). This study focused on depression among college freshmen and used a longitudinal tracking method to refine the empirical field of this theory. Future research will continue to explore the adaptation of college freshmen in more detail.

Secondly, Study 2 examined the bidirectional predictive relationship between state depression and PSS. The results showed that state depression on one day negatively predicted PSS on the following day, and vice versa. That is, state depression and PSS exhibited a bidirectional negative predictive relationship over the 7-day period. This finding validates hypotheses H2 and H4. It should be noted that although the effect size is small, its statistical significance and theoretical consistency provide empirical evidence for the short-term protective role of social support. This can be explained by social withdrawal theory, which suggests that while social support may not immediately or drastically reduce depression, it can consistently exert small, cumulative positive effects on a day-to-day basis. As Funder and Ozer (2019) emphasized, even weak effects in psychological research can have significant practical implications over the long term and through repeated interactions, reflecting the dynamic coupling between social interaction and emotional experience.

Specifically, individuals with depression tend to avoid social interactions as much as possible, reducing their social opportunities, which in turn lowers their perceived level of social support (Wood et al., 2022), consistent with previous findings (Brown et al., 2023). Depressive states may cause college freshmen to have a diminished ability to perceive and utilize social support, further lowering their PSS levels. For example, college freshmen in a depressive state, upon entering a new environment, may ignore the care and support offered by those around them. Even if they recognize the existence of such support, they may struggle to effectively use these resources to alleviate their psychological distress. This decline in perception and utilization further exacerbates feelings of loneliness and helplessness, making individuals more likely to fall into depressive moods, thereby creating a negative cycle (Zhou and Liu, 2025). Depressed individuals often tend to overestimate others’ negative impressions of themselves, a bias that can predict social anxiety in adolescents (Zhang and Zhou, 2018), which in turn affects their perception of social support. Social support can mitigate the negative effects of life stress on depression. In the face of stressors, PSS can reduce feelings of helplessness and increase confidence in facing difficulties. Therefore, a decrease in PSS further exacerbates the severity of depression. For college freshmen, encountering new interpersonal relationships makes them more prone to social anxiety and self-isolation. They may fail to perceive the social support provided by those around them, leading to further self-doubt and impacting their adaptation to university life (Liao et al., 2025). Li P. et al. (2024) and Li Y. et al., 2024 in their study on the relationship between childhood emotional abuse and daily depressive moods, confirmed through a 14-day diary method that PSS could serve as a factor explaining the formation mechanism of depression. This study expands the breadth and depth of research on college freshmen’s adaptation and constructs a bidirectional predictive model of depression and PSS, further clarifying their mutual influence.

This study combined three months of longitudinal tracking with a seven-day diary method to reveal the bidirectional dynamic relationship between depression and PSS among college freshmen. Theoretically, the study integrates existing research and relevant theories, emphasizing individuals’ subjective perceptions of social support. It provides a more comprehensive framework for understanding the psychological adaptation mechanisms of college freshmen. Practically, the bidirectional predictive model constructed in this study captures both long-term trends and daily fluctuations, moving beyond the one-way assumptions of previous research. It offers an operational tool for the early identification of depression risk and optimizing psychological interventions. Based on this model, researchers can implement targeted interventions, such as psychological counseling and cognitive-behavioral adjustments, to enhance students’ PSS and psychological resilience, thereby reducing the incidence of depression.

In terms of methodological innovation, this study employed both longitudinal tracking and diary methods, which not only reduced recall bias but also captured individual fluctuations. Furthermore, it revealed individual differences, making the conclusions more predictive, verifiable, and targeted. This approach provides new insights and methodological foundations for future theoretical expansion and empirical interventions. Other research has suggested that factors such as communication styles, labeling, and cultural attitudes may influence the provision and perception of support (Boldrini et al., 2024). Future studies will consider additional influencing factors and broader psychological issues to further promote the mental health of college freshmen (Lo Buglio et al., 2022).

Despite its contributions, this study has several limitations that need to be addressed. First, both the three-month longitudinal tracking in Study 1 and the seven-day diary method in Study 2 had relatively short durations. However, existing research has confirmed the feasibility of these methods (de Ridder et al., 2021; Li P. et al., 2024; Li Y. et al., 2024), and the consistency between the three-month and seven-day results in this study supports the validity of the conclusions. Future studies should further refine and improve these methods. Second, although the scales used in this study have demonstrated good reliability and validity, the data collection method relied on self-reports, which may introduce some biases. Future research could employ more rigorous methods, such as combining self-report and other-report measures, to explore the relationship between depression and PSS from a more objective perspective. Third, the majority of participants in this study were female, which may limit the external validity of the results. Previous studies have shown that gender differences can affect the expression of depressive symptoms (Tanaka et al., 2022) as well as the perception and utilization of social support (Kuehner, 2017). Therefore, future research should aim for a more balanced gender ratio among participants to enhance the representativeness and external validity of the findings. Fourth, existing research has indicated that socioeconomic status (SES) may influence an individual’s PSS and depressive symptoms. Individuals with higher SES typically possess greater social resources, stronger social networks, and lower levels of psychological distress (Adler et al., 1994; Matthews and Gallo, 2011; Conger et al., 2010). The absence of SES measures in the current study may limit the generalizability of the findings. However, since all participants in this study were first-year university students, their overall SES levels are relatively homogeneous. Future research could consider incorporating samples with more diverse socioeconomic backgrounds and extend beyond the first-year university student population to enhance the external validity of the findings. Finally, this study did not account for the “week effect” in the diary data. Previous research has shown that individuals’ emotional states and perceptions of social support may exhibit systematic fluctuations over the course of the week, which can be linked to changes in academic pressure, social activities, and rest rhythms (Ryan et al., 2010; Stone et al., 2006). Future research should consider the week effect to better understand the dynamic relationship between social support and emotional fluctuations.

## 5 Conclusion

This study combined longitudinal tracking and diary methods to explore the bidirectional relationship between PSS and depressive symptoms across short-term and long-term timeframes.

The results indicate a bidirectional predictive relationship between the two variables.

## Data Availability

The raw data supporting the conclusions of this article will be made available by the authors, without undue reservation.
